# Macromolecular juggling by ubiquitylation enzymes

**DOI:** 10.1186/1741-7007-11-65

**Published:** 2013-06-25

**Authors:** Sonja Lorenz, Aaron J Cantor, Michael Rape, John Kuriyan

**Affiliations:** 1 Department of Molecular and Cell Biology, University of California, Berkeley, CA 94720, USA; 2 California Institute for Quantitative Biosciences, University of California, Berkeley, CA 94720, USA; 3 Department of Chemistry, University of California, Berkeley, CA 94720, USA; 4 Howard Hughes Medical Institute, University of California, Berkeley, CA 94720, USA; 5 Physical Biosciences Division, Lawrence Berkeley National Laboratory, Berkeley, CA 94720, USA

## Abstract

The posttranslational modification of target proteins with ubiquitin and ubiquitin-like proteins is accomplished by the sequential action of E1, E2, and E3 enzymes. Members of the E1 and E3 enzyme families can undergo particularly large conformational changes during their catalytic cycles, involving the remodeling of domain interfaces. This enables the efficient, directed and regulated handover of ubiquitin from one carrier to the next one. We review some of these conformational transformations, as revealed by crystallographic studies.

## 

To catalyze multistep reactions some metabolic enzymes undergo major structural rearrangements. By disassembling the interfaces between domains and then reassembling them differently, these enzymes create distinct active sites and recognize multiple substrates sequentially. Having one enzyme that can restructure itself to carry out two or more steps in sequence is presumably more efficient than parsing out the tasks to separate enzymes and also reduces the risk of losing intermediate products, particularly those that are chemically labile. Catherine Drennan and colleagues recently introduced the term ‘molecular juggling’ [1] to describe the large structural rearrangements of enzymes involved with B_12_-dependent methyl transfer reactions [1-3]. One of us (JK) encountered a similar phenomenon in the early 1990s when studying the bacterial thioredoxin reductase enzyme [4-6]. Other examples of molecular juggling are provided by the ANL (acyl-CoA synthetases, non-ribosomal peptide synthetase adenylation domains, and luciferase) superfamily of adenylating enzymes (for review, see [7]). The last decade has seen a dramatic expansion in structural information for a set of enzymes that control the addition of ubiquitin, a small protein, to target proteins. This new structural window into ubiquitylation enzymes has revealed them to be molecular jugglers of a most sophisticated kind, as noted for one class of these enzymes by Christopher Lima and coworkers [8]. In this review we survey what we have learned from crystallographic studies about the large conformational changes in ubiquitylation enzymes.

Ubiquitylation controls protein trafficking and degradation as well as complex signaling pathways, such as DNA repair and immune responses (for reviews, see [9,10]). The diverse physiological roles of ubiquitin originate, at least in part, from the many ways by which it can be attached to target proteins. Target proteins may be tagged with one or several individual ubiquitin molecules or with polymeric ubiquitin chains. These chains are linked through isopeptide bonds between the carboxyl terminus of one ubiquitin molecule and a primary amino group on another. Ubiquitin contains seven lysine residues and a free amino terminus, so the chains can have many different topologies, depending on the enzymes involved in assembling them. The various types of ubiquitin modifications are recognized by different downstream effectors in the cell and trigger distinct functional outcomes (for reviews, see [11,12]). Further diversity arises from the existence of several ubiquitin-like protein modifiers, such as SUMO (small ubiquitin-like modifier) and NEDD8 (neural precursor cell expressed, developmentally down-regulated 8) that utilize their own enzymatic machineries and are associated with distinct physiological responses (for review, see [13]). We shall draw on structural information from studies on all three of these modifiers, and will, where appropriate, refer to ubiquitin and ubiquitin-like proteins collectively as ‘Ubl’.

Ubiquitylation is accomplished through a catalytic cascade involving ubiquitin-activating enzymes (E1), ubiquitin-conjugating enzymes (E2), and ubiquitin ligases (E3) (for review, see [14]). The human proteome contains two E1 enzymes [15-18], approximately 40 E2 enzymes [19], and over 600 E3 enzymes [20], the combination of which accounts for the large variety of ubiquitin modifications. To transfer ubiquitin from one carrier to the next one, ubiquitylation enzymes sequentially form and reorganize protein-protein interfaces. We thus use the term ‘macromolecular juggling’ to describe these actions.

E1 enzymes catalyze the formation of a thioester-linked complex between ubiquitin and E2 enzymes (for review, see [14]) (Figure 1a). This process begins by activation of the carboxyl terminus of ubiquitin by adenylation, followed by a thioesterification reaction in which ubiquitin is conjugated to a cysteine residue at the active site of the E1 enzyme. Ubiquitin is then transferred to the active site cysteine of an E2 enzyme in a trans-thioesterification reaction.

**Figure 1:**
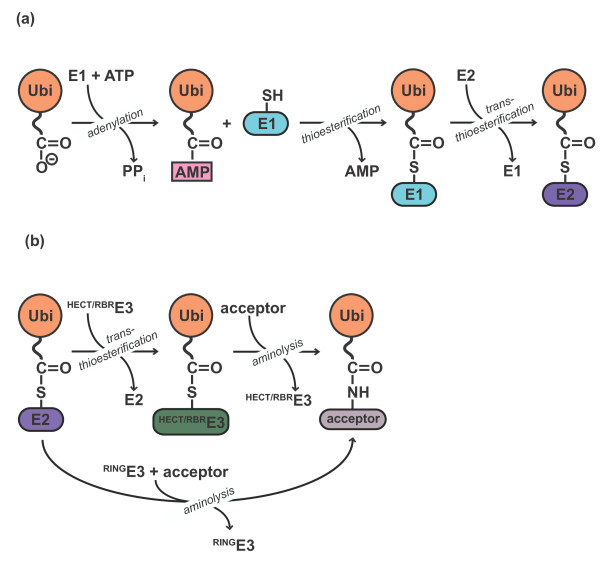
**Ubiquitylation is a multistep reaction. (a)** E1 enzymes use ATP to activate the carboxyl terminus of ubiquitin (Ubi) as a high-energy anhydride (Ubi-AMP). The E1 active site cysteine then attacks the adenylated ubiquitin to form a thioester intermediate. Subsequently, the active site cysteine of the E2 receives ubiquitin via trans-thioesterification. **(b)** E3 enzymes catalyze the formation of an isopeptide bond between the ubiquitin carboxyl terminus and a primary amino group of an acceptor. The acceptor can be a target protein (mono-ubiquitylation/ubiquitin chain initiation) or another ubiquitin molecule (ubiquitin chain elongation). Catalysis by HECT- and RBR-type E3 enzymes proceeds through an intermediate, in which the ubiquitin carboxyl terminus is thioester-linked to a cysteine residue at the active site of the E3, followed by aminolysis of the thioester. In contrast, RING-type E3s catalyze direct transfer of ubiquitin from the E2 active site cysteine to amino groups on the acceptor.

The transfer of ubiquitin from ‘charged’ E2 enzymes onto target proteins is mediated by enzymes of the E3 family. The common outcome of E3-catalyzed reactions is an isopeptide linkage between the carboxyl terminus of ubiquitin and a primary amino group on a target protein. However, E3 enzymes vary significantly in size and subunit composition and follow different mechanisms (for reviews, see [21,22]): RING (really interesting new gene) domain-containing E3 enzymes and the related U-box E3s interact with charged E2 enzymes and target proteins simultaneously and facilitate direct ubiquitin transfer from the E2 onto the target protein (Figure 1b). In contrast, the mechanism of HECT (homologous to the E6-AP C-terminus) domain-containing E3 enzymes includes an additional trans-thioesterification step, in which ubiquitin is linked to a catalytic cysteine on the E3. The resulting charged E3 then transfers ubiquitin to the target protein (Figure 1b). A combination of both mechanisms is used by the RING-in-between-RING (RBR) family of E3s. Like HECT E3s, RBRs contain a catalytic cysteine and form a thioester-linked intermediate with ubiquitin before passing it on to the target protein [23] (Figure 1b). However, they also utilize a canonical RING domain to recruit the charged E2 enzyme (for review, see [22]).

As revealed by a growing body of structural data, E1 and E3 enzymes undergo striking remodeling of domains during their catalytic cycle. In contrast, most E2 enzymes are relatively small, single-domain proteins and do not utilize large-scale structural changes for Ubl transfer [24-26].

We describe the conformational changes of E1 and HECT-type E3 enzymes in the first part of this review. Unlike E2 or RING-type E3 enzymes, these two classes of ubiquitylation enzymes catalyze multistep reactions. Structural rearrangements allow these enzymes to bind multiple sequential substrates and process them in distinct active sites. As reviewed elsewhere [27,28], structural flexibility has also been observed in cullin-RING ligases, a group of multisubunit RING-type E3 enzymes, which catalyze one-step ubiquitin transfer reactions.

In the second part, we describe conformational changes that are involved in modulating the activity of ubiquitylation enzymes. Such regulatory rearrangements are perhaps best understood for E3 enzymes. We have chosen to focus on the way structural flexibility is exploited in the regulation of the single-subunit RING E3 Cbl [29,30].

## E1 enzymes reorganize domains during their catalytic cycle

The catalytic mechanism of E1 enzymes includes three reactions that require distinct active site environments: (i) adenylation, (ii) thioesterification, and (iii) trans-thioesterification. Our current understanding of the conformational changes that canonical E1 enzymes undergo during catalysis stems primarily from crystallographic studies carried out by the groups of Brenda Schulman [31-36], Christopher Lima [8,37] and Hermann Schindelin [38]. These studies were performed with different E1 enzymes that operate on ubiquitin and its close relatives, SUMO and NEDD8, respectively. All three of these E1s appear to follow a conserved general mechanism of catalysis, and because of their related domain structures they are classified as ‘canonical’ (for review, see [39]). In contrast, ‘non-canonical’ E1 enzymes, such as the one that is specific for the autophagy-related Ubls ATG8 and ATG12 have distinct structures and mechanisms [40-43].

**Figure 2:**
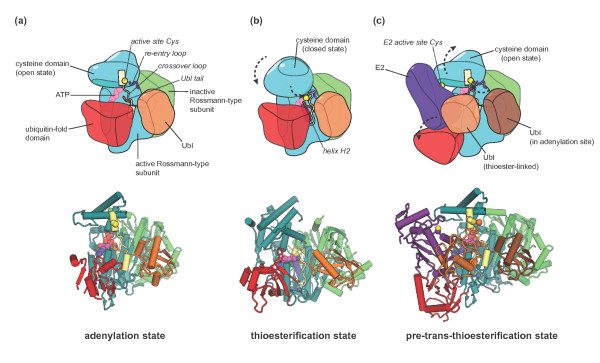
**Conformational rearrangements in E1 enzymes.** Cartoon representations of distinct states in the catalytic cycle of canonical E1 enzymes. **(a)** The adenylation state based on the crystal structure of NAE1-UBA3 in complex with NEDD8 and ATP/Mg^2+^ [PDB: 1R4N] [32]. The carboxy-terminal tail of the Ubl is in the adenylation site of the active Rossmann-type subunit of the E1, ready to nucleophilically attack the α-phosphate of the ATP to form the Ubl-AMP intermediate. The catalytic cysteine residue in the E1 cysteine domain is part of an α-helix and is removed from the adenylation site, giving rise to an open conformation of the cysteine domain. **(b)** The thioesterification state as seen in a crystal structure of SAE1-UBA2 and SUMO covalently coupled to an AMP analogue that mimics the tetrahedral intermediate generated during thioesterification [PDB: 3KYD] [8]. Mediated by large conformational changes in the crossover and re-entry loops, the cysteine domain is rotated with respect to the Rossmann-type subunits. The helix containing the active site cysteine seen in (a) has melted. In this closed conformation of the cysteine domain, the catalytic cysteine nucleophile is in position to attack the adenylated carboxyl terminus of the Ubl. The positive dipole of helix H2 in the active Rossmann-type subunit (colored purple) is thought to favor this reaction [8]. **(c)** The trans-thioesterification state as represented by a crystal structure of NAE1-UBA3 thioester-linked to NEDD8 and in complex with an additional NEDD8 molecule, an E2 enzyme (Ubc12) and ATP/Mg^2+^[35]. The cysteine domain of the E1 adopts an open orientation similar to the adenylation state (a), but now holds the carboxyl terminus of the thioester-linked Ubl close to the E2 active site (a Cys-to-Ala mutant of the E2 was used in this study (see text)). The ubiquitin-fold domain has swung away from its position in the previous states (a,b) to accommodate the E2 and contributes to E2 binding. In (a,c) domains found in NAE1-UBA3 but not in SAE1-UBA2 were omitted for clarity. To see a rendition of a dynamic transition between the structures shown in the lower panels of (a-c), see Additional file 1. As noted in the movie legend, the details of the trajectory linking individual structures is not realistic and is simply meant to illustrate the nature of the conformational changes rather than identify the nature of the transition pathway.

Canonical E1 enzymes contain two Rossmann-type folds (either as domains within the same polypeptide chain or as separate subunits in the context of a heterodimer), a domain containing the catalytic cysteine (the cysteine domain), and a ubiquitin-fold domain [31,37,38] (for review, see [39]) (Figure 2). The two Rossmann-type subunits are functionally distinct and form a quasi-symmetric dimer that catalyzes the modification of a single Ubl molecule at a time. Only one subunit, the ‘active’ Rossmann-type subunit, binds the ATP that is required for adenylation of the terminal carboxyl group of the Ubl. Topologically, the cysteine domain is inserted into the active Rossmann-type subunit. The two connections between the cysteine domain and the Rossmann-type subunit are known as the ‘crossover’ and ‘re-entry’ loops and have an important role in enabling the movement of the cysteine domain during catalysis [8]. The ubiquitin-fold domain contributes to the recruitment of the E2 enzyme onto which the E1-bound Ubl is transferred in a trans-thioesterification reaction [31,34,37,38].

An impressive range of crystallographic snapshots of various catalytic stages of canonical E1 enzymes have outlined the conformational dynamics in this enzyme family [8,31,32,35,37,38,44]. Below, we describe the major structural changes that facilitate the three chemically distinct reaction steps.

##  Macromolecular juggling by ubiquitylation enzymes

## The E1 cysteine domain adopts an open conformation during Ubl adenylation

E1 enzymes initially activate the carboxyl terminus of their Ubl substrates by adenylation. In this reaction, the terminal carboxylate of the Ubl attacks the α-phosphate of ATP bound to the active Rossmann-type subunit, releasing pyrophosphate and generating a Ubl-AMP conjugate.

The first structural insights into Ubl recognition by E1 enzymes came indirectly, from studies on their bacterial ancestors, MoeB and ThiF. These proteins participate in the biosynthesis of molybdenum cofactor and thiamine by adenylating the carboxyl terminus of the ubiquitin-fold proteins MoeD and ThiS, respectively [45-51]. Unlike canonical E1 enzymes, MoeB and ThiF contain two catalytically active Rossmann-type subunits [49-51]; the structural details of their binding to ubiquitin-fold proteins are, however, conserved (for review, see [52]).

Ubl recognition by E1 enzymes involves hydrophobic contacts between residues in the active Rossmann-type subunit and a hydrophobic patch on the globular core of the Ubl [32,37,38]. The carboxy-terminal flexible tail of the Ubl protrudes into a shallow cleft on the E1 surface and points toward the ATP binding pocket (Figure 2a), where it is clamped tightly by the crossover loop connecting the cysteine domain and the active Rossmann-type subunit. The ATP binding pocket itself is solvent-accessible, which allows the pyrophosphate product of the adenylation reaction to diffuse out, thereby reducing back-reactions. Residues critical for ATP/Mg^2+^ binding and catalysis are highly conserved [31,32,37,38,49,51], but contacts between the Ubl tail and the crossover loop vary across different E1 enzymes, and contribute to their specificity for particular Ubls [32,36,53-55]. Ubiquitin- and NEDD8-specific E1 enzymes form additional electrostatic contacts with their Ubls, which are mediated by unique domains found in these enzymes [32,38].

During the adenylation reaction, the cysteine domain of the E1 adopts an open conformation in which it makes few contacts with the active Rossmann-type subunit, and the catalytic cysteine residue is separated from the carboxyl terminus of the bound Ubl by over 30 Å [8,32,37,38]. In the subsequent thioesterification reaction, however, the catalytic cysteine residue is linked to the Ubl carboxyl terminus. To accomplish this, the E1 enzyme must either allow release of the Ubl and diffusion towards the catalytic cysteine or, as is the case, major domain rearrangements around the bound Ubl.

## The E1 cysteine domain adopts a closed conformation for thioesterification

How E1 enzymes switch between conformations that facilitate adenylation and thioesterification, respectively, was revealed by Christopher Lima, Derek Tan and colleagues. They used a chemical strategy to trap a covalent complex, in which the SUMO-specific E1 enzyme (SAE1-UBA2), SUMO and an AMP analogue are linked covalently to each other in such a way that the active site environment mimics the environment around the tetrahedral intermediate that is formed during the nucleophilic attack by the catalytic cysteine of the E1 on the adenylated SUMO tail (Protein Data Bank (PDB) accession [PDB: 3KYD]) [8].

The E1 cysteine domain in this complex is rotated by approximately 130° with respect to the open state, now adopting a ‘closed’ conformation, in which it forms extensive contacts with the active Rossmann-type subunit (Figure 2b). Large conformational rearrangements also occur in the crossover and re-entry loops connecting the cysteine domain to the active Rossmann subunit, and several structural elements in the cysteine domain and in both Rossmann-type subunits become disordered. In particular, the region of the cysteine domain that bears the catalytic cysteine is helical in the open state, but becomes extended in the closed conformation, enabling the cysteine to reach into the adenylation pocket.

How does the active site environment in the closed conformation of the cysteine domain stimulate the thioesterification reaction? In principle, one would expect the presence of basic residues that could promote the deprotonation of the cysteine nucleophile. Surprisingly, however, the active site environment in the closed state does not contain any side chains that could potentially act as general acid/base catalysts. Instead, it places the catalytic cysteine residue near the amino-terminal end of helix H2 of the active Rossmann-type subunit (Figure 2b). Lima and coworkers propose that the positive H2 helix dipole electrostatically stabilizes the transition states of both the adenylation and the thioesterification reactions [8].

## Ubl transfer to the E2 requires reorientation of the ubiquitin-fold domain

Before the Ubl protein is passed from the catalytic cysteine of the E1 to that of the E2, a second Ubl protein is adenylated by the E1 [56,57]. The E1 enzyme thus becomes loaded with two Ubl proteins, one that is thioester-linked to the catalytic cysteine of the E1 and a second one bound non-covalently in the adenylation site. Interestingly, binding of the second Ubl protein at the adenylation site of the E1 facilitates the transfer of the thioesterified Ubl protein to the E2 enzyme [58].

The structural basis for this coupling between the two Ubls was revealed by Brenda Schulman and colleagues, who solved a crystal structure of the doubly loaded state of the NEDD8-specific E1 (NAE1-UBA3) in complex with a cognate E2 enzyme (Ubc12) [PDB: 2NVU] [35]. To trap this state and prevent NEDD8 transfer onto the E2 enzyme, the catalytic cysteine residue of the E2 was replaced by alanine. In this structure the E1 cysteine domain adopts an open conformation, thereby removing the thioester-linked Ubl from the adenylation site, as required for binding of the second Ubl protein in this site. To accommodate the re-oriented thioester-linked Ubl and the E2 enzyme, the ubiquitin-fold domain of the E1 undergoes a large outward swing with respect to the Rossman-type subunits (Figure 2c).

The E2 enzyme is recognized in tripartite fashion by the doubly loaded E1 enzyme [35]: one set of interactions is contributed by the ubiquitin-fold domain of the E1, a second set is provided by the active Rossmann-type subunit, and the third involves the Ubl that is thioester-linked to the active site of the cysteine domain. Upon Ubl transfer from the E1 catalytic cysteine to the E2, one face of the tripartite interaction between the E1 and the E2 is lost: the Ubl, now linked to the E2, no longer provides a covalent tether to the E1. The resulting decrease in affinity between E1 and E2 presumably facilitates a backward swing of the ubiquitin-fold domain of the E1, thereby enabling product release. The conformational switch of the ubiquitin-fold domain, together with the tripartite, Ubl-assisted nature of E2 binding, thus adds directionality to the trans-thioesterification reaction. In line with this mechanism, mutations that restrict the freedom of movement of the ubiquitin-fold domain decrease the efficiency of Ubl transfer onto the E2 [34,38]. Contacts between the ubiquitin-fold domain and the E2 enzyme also contribute to the specificity of E1 enzymes for particular Ubls [16,59-61] (for review, see [39]).

Notably, the crystallographic snapshot of doubly loaded E1 in complex with the E2 leaves an estimated approximately 20 Å gap between the active site cysteine residues of the E1 and the E2 [35], indicating that trans-thioesterification occurs in another, yet uncharacterized, conformation.

## HECT E3 enzymes require structural plasticity for catalysis

**Figure 3:**
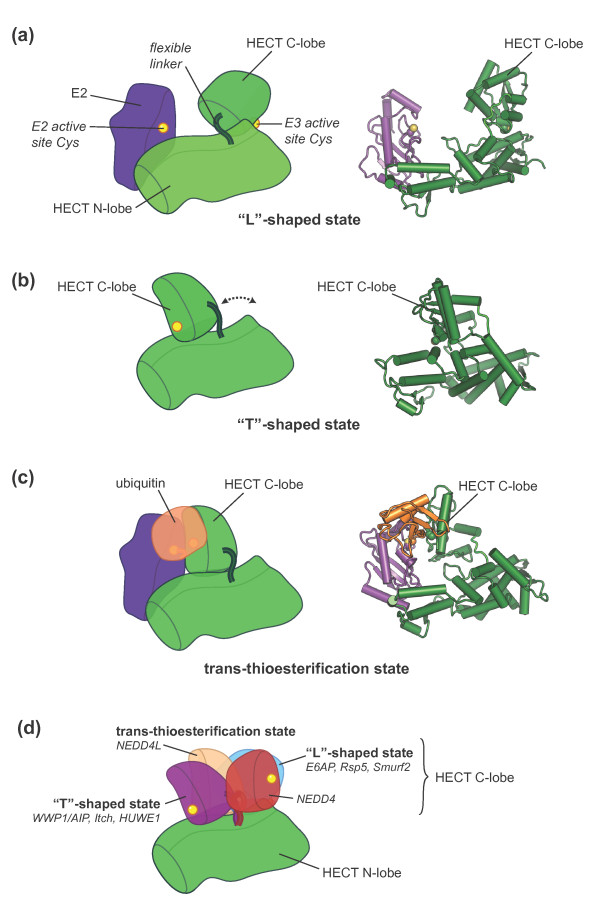
**Swinging domains in HECT E3 enzymes.** Cartoon representations of crystal structures of various HECT domains. **(a)** Open, ‘L’-shaped conformation of E6AP (E3) in complex with UbcH7 (E2) [PDB: 1C4Z] [62], **(b)** closed, ‘T’-shaped conformation of WWP1/AIP [PDB: 1ND7] [63], and **(c)** trans-thioesterification complex of NEDD4L with a ubiquitin-E2 (UbcH5B) conjugate [PDB:3JVZ] [64]. In (c) the E2 active site cysteine was mutated to serine (colored yellow in our representation), resulting in an *oxy*-ester linkage with ubiquitin in lieu of the native thioester. **(d)** Distinct classes of C-lobe orientations based on the crystal structures of various HECT domains (WWP1/AIP [PDB: 1ND7], Itch [PDB: 3TUG], HUWE1 [PDB: 3G1N, 3H1D], NEDD4L [PDB: 2ONI, 3JVZ], E6AP [PDB: 1C4Z], Rsp5 [PDB: 3OLM], Smurf2 [PDB: 1ZVD], NEDD [PDB: 2XBB]). A second unique C-lobe orientation observed for NEDD [PDB: 2XBF] could not be displayed for clarity. In our representation the HECT N-lobes are superimposed and only one of them is displayed. Binding partners, such as E2 enzymes or ubiquitin, found in some of the structures are not displayed.

Once ubiquitin has been linked to the E2 enzyme, an E3 enzyme catalyzes the transfer of ubiquitin to a target protein. For E3s in the HECT and RBR families, this process involves the formation of an intermediate in which ubiquitin is thioester-linked to a catalytic cysteine residue of the E3 (Figure 1b). Like E1 enzymes, these E3 enzymes thus catalyze multistep reactions. Nikola Pavletich and coworkers [62] predicted considerable structural flexibility in HECT E3 enzymes when they determined the first crystal structure of a HECT family member, the HECT domain of E6AP in complex with the E2 enzyme UbcH7 [PDB: 1C4Z]. HECT domains (approximately 40 kDa) consist of two lobes, a large amino-terminal or N-lobe containing the E2 binding site and a smaller carboxy-terminal or C-lobe bearing the catalytic cysteine. In the E2-bound state, the two lobes of E6AP were found to adopt an open, ‘L’-shaped conformation, giving rise to a >40 Å gap between the active site cysteine residues of the E2 and the E3 (Figure 3a). Transfer of ubiquitin between these sites was thus expected to involve conformational rearrangements.

Indeed, the two lobes are fundamentally reorganized with respect to each other in a crystal structure of the HECT domain of WWP1/AIP solved by Joseph Noel and colleagues [PDB: 1ND7] [63]. This HECT domain adopts a closed conformation, reminiscent of the letter ‘T’ (Figure 3b). A flexible hinge region connecting the two HECT lobes enables this remarkable rearrangement, and mutations that restrict conformational freedom in this region inhibit the ubiquitylation activity of WWP1/AIP *in vitro*, attesting to the functional importance of structural flexibility in this enzyme [63]. The transition from the open to the closed state of the HECT domain is expected to bring the active sites of the E3 and a bound E2 closer to each other. However, a remaining gap of approximately 17 Å between the catalytic centers of E2 and E3 (estimated from the crystal structure of WWP1/AIP and modeling of the E2 according to the E6AP-UbcH7 structure [62]) indicated additional conformational changes yet to be uncovered that would allow trans-thioesterification.

Key insights into these structural changes came from Brenda Schulman’s group, who determined a crystal structure of the HECT-domain of NEDD4L in complex with a thioester-linked E2-ubiquitin conjugate [PDB: 3JVZ] [64]. The complex adopts a compact conformation, in which the HECT C-lobe is rotated markedly compared to previous structures, and makes contacts with E2-bound ubiquitin (Figure 3c). This interaction is mediated by a conserved hydrophobic surface on the C-lobe of the E3 and appears to tether the C-lobe in proximity to the E2 - as was hypothesized by Pavletich and colleagues [62]. A remaining gap of approximately 8 Å between the catalytic centers of E2 and E3 in this structure could readily be closed by additional small rotations around the flexible hinge region, yielding a functional trans-thioesterification intermediate.

Crystal structures of several other HECT domains are now available, including ‘open-like’ states of Rsp5 [PDB: 3OLM] [65] and Smurf2 [PDB: 1ZVD] [66], closed states of Itch [PDB: 3TUG] and HUWE1 [PDB: 3G1N, 3H1D] [67], two unique states of NEDD4 [PDB: 2XBF, 2XBB] [68], and NEDD4L in an *apo* conformation that resembles its trans-thioesterification state with ubiquitin-charged E2 [PDB: 2ONI] (Figure 3d). Taken together, these HECT domain structures show a considerable variation in the relative orientations of N- and C-lobes, indicating that dynamic rearrangements are a common feature in the HECT E3 family. Interestingly, the same is true for HECT E3-like proteins found in bacterial pathogens [69-71]. These bacterial proteins can interact with eukaryotic E2 enzymes *in vitro* and are thought to ‘hijack’ the ubiquitylation system upon delivery into the cytosol of the eukaryotic host, thereby regulating host inflammatory responses [69].

The structural plasticity of HECT-like E3 enzymes in both eukaryotic and prokaryotic systems is consistent with a functional role for this level of flexibility during catalysis. It is possible that domain movements of HECT E3s are implicated in the iterative binding and release of E2 enzymes or the repositioning of ubiquitin substrates, as might be required during the formation of a ubiquitin chain [63,64]. The mechanism of ubiquitin chain formation by HECT E3 enzymes, however, remains controversial and is likely to vary between enzymes [72,73].

## The structural flexibility of E3 enzymes is harnessed for their regulation

Since E3 enzymes require flexibility during their catalytic cycle, they can be regulated by processes that restrict their flexibility and lock them in particular conformations. The HECT E3s Itch and Smurf2, for example, are negatively regulated through intra- and intermolecular interactions between their catalytic HECT domains and preceding WW and C2 domains, respectively [74,75]. Autoinhibition is relieved upon phosphorylation in the amino-terminal part of Itch [74] and binding of Smurf2 to the adaptor protein Smad7, respectively [75]. Autoinhibitory domain interactions have also been identified for E3 enzymes in the RBR [76] and RING families (for review, see [77]).

A structural mechanism for regulation of RING-type E3 enzymes by posttranslational modifications was first described for the multisubunit cullin-RING ligases. As suggested by biochemical studies indicative of conformational rearrangements [78,79], covalent attachment of the Ubl NEDD8 to the cullin subunit results in a dramatic re-orientation of the RING domain that places the bound E2 adjacent to the substrate, thereby activating the ligase [80]. This conformational switch is harnessed by various cellular effectors that restrict the conformational flexibility of cullin-RING ligases (for reviews, see [28,77]). Other RING-type E3 enzymes are regulated through conformational changes that affect their oligomerization state, as demonstrated for inhibitor of apoptosis proteins (IAPs) [81,82] and tumor necrosis factor receptor-associated factor (TRAF6) [83]. We will focus here on the recently elucidated role of phosphorylation-induced structural rearrangements in the regulation of the single-subunit RING E3 enzyme Cbl.

## Phosphorylation triggers regulatory domain rearrangements in CBL proteins

**Figure 4:**
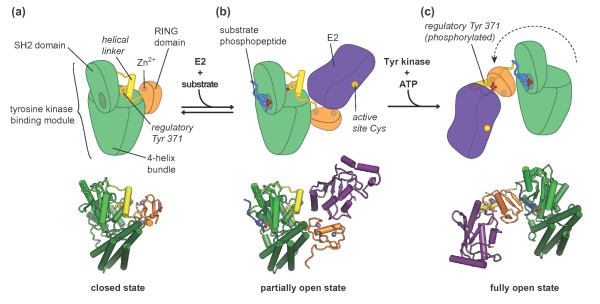
**Regulatory rearrangements in Cbl proteins. (a)** ‘Closed’ conformation of Cbl based on the crystal structure of the *apo* c-Cbl amino-terminal region, comprising the tyrosine kinase binding module, the helical linker region, and the RING domain [PDB: 2Y1M] [29]. The regulatory tyrosine, Y371, located in the helical linker region, is buried in a hydrophobic core formed by the SH2 domain and the four-helix bundle in the tyrosine kinase binding module. **(b)** ‘Partially open’ conformation of Cbl based on the co-crystal structure of c-Cbl amino-terminal region with a ZAP70-derived phosphopeptide and the E2 enzyme UbcH7 [PDB: 1FBV] [91]. Phosphopeptide binding induces a shift in the SH2 domain that perturbs the interface between the helical linker and the tyrosine kinase binding module, probably favoring dissociation of the RING domain from the tyrosine kinase binding module and thus increasing the accessibility of the E2 binding surface. **(c)** ‘Open’ conformation of Cbl based on the co-crystal structure of phosphorylated c-Cbl bound to a ZAP7-derived phosphopeptide and UbcH5B [PDB: 4A4C] [29]. The phosphorylated regulatory tyrosine, Tyr371, interacts with residues in the E2 binding surface of the RING domain. The RING domain is situated on the opposite side of the tyrosine kinase binding module compared to (b).

Cbl proteins (c-Cbl, Cbl-b and Cbl-c) are a family of single-subunit RING E3 enzymes that ubiquitylate receptor and non-receptor tyrosine kinases and thereby regulate both the trafficking and the degradation of these kinases (for reviews, see [84,85]). Members of the Cbl family share a conserved amino-terminal tyrosine kinase binding module that includes an SH2 (Src homology 2) domain, as shown by Michael Eck and colleagues [86,87], and is connected to the RING domain through a helical linker. The SH2 domains of Cbl proteins bind to phosphorylated tyrosine residues on substrates, including receptor-tyrosine kinases such as the epidermal growth factor receptor [88] and the T-cell receptor-associated tyrosine kinase Zap70 [86,89]. The RING domain recruits the E2 enzyme (for review, see [90]). The first view of how RING domains recognize E2 enzymes was provided by Nikolai Pavletich and coworkers [91], who determined the crystal structure of the tyrosine kinase binding module, linker helix and RING domain of c-Cbl in complex with a phosphorylated Zap70-derived peptide and the E2 enzyme UbcH7 [PDB: 1FBV] (Figure 4b). However, this structure showed a large gap between the E2 active site and the substrate peptide, and with no information on the spatial orientation of the target protein with respect to this peptide, it remained unclear how ubiquitin is transferred to the target. Moreover, although the co-crystal structure of c-Cbl and UbcH7 represents a canonical E2-E3 complex, UbcH7 and c-Cbl do not form an active and physiologically relevant E2-E3 pair [92,93]. Another structural puzzle arose from the discovery that phosphorylation of Cbl proteins in the linker helix region increases their ubiquitin ligase activity [88,94-96]. Phosphorylation is incompatible with the conformation observed in the first crystal structures because the modification site, Tyr371 in c-Cbl, is buried at the interface of the tyrosine kinase binding module and the helical linker region (Figure 4b).

Two independent studies have recently shed light on this discrepancy and have revealed the central role of conformational plasticity in Cbl regulation. Danny Huang and colleagues [29] presented three crystal structures of a c-Cbl fragment comprising the tyrosine kinase binding module, the helical linker region and the RING domain: (i) the *apo* form [PDB: 2Y1M], (ii) c-Cbl bound to a phosphorylated Zap70-derived peptide [PDB: 2Y1N], and (iii) phosphorylated c-Cbl in a ternary complex with the phosphorylated Zap70-derived peptide and the E2 enzyme UbcH5B [PDB: 4A4B]. Fuyuhiko Inagaki and coworkers [30] provided nuclear magnetic resonance (NMR) and small-angle X-ray scattering (SAXS) data on Cbl-b supporting the existence of distinct conformations in solution and highlighting the flexible nature of Cbl family proteins.

In the absence of substrate, Cbl favors a compact, autoinhibited, ‘closed’ conformation, in which contacts between the tyrosine kinase binding module and the RING domain obstruct the E2 binding site [29,30] (Figure 4a). Binding of substrate peptide to the SH2 domain perturbs the closed conformation, which releases the RING domain and opens up the E2 binding site [29,30]. This ‘partially open’ state, as represented by the previous c-Cbl-UbcH7 co-crystal structure [91], shows a tight association between the linker helix region and the tyrosine kinase binding module (Figure 4b). In solution, however, the partially open state is in a dynamic equilibrium with other conformations that make the regulatory tyrosine residue accessible [30]. Phosphorylation at this site stabilizes a ‘fully open’ state, in which the helical linker region is completely dissociated from the tyrosine kinase binding module and instead makes contact with the RING domain [29,30] (Figure 4c). The phosphorylated tyrosine on the linker helix forms ionic interactions with lysine residues on the RING domain, whose positive charge might otherwise repel the positively charged binding surface of the E2 [30]. The RING domain also undergoes a dramatic re-orientation relative to the tyrosine kinase binding module, which significantly reduces the distance between the E2 active site and the bound substrate peptide [29]. Taken together, the conformational opening thus increases the affinity of Cbl proteins for E2 enzymes as well as their catalytic efficiency of ubiquitin transfer.

While these studies reveal how posttranslational modifications and allosteric effects can induce a shift in the conformational equilibrium of Cbl proteins, it is not the end of the story. For c-Cbl and Cbl-b, dimerization through their carboxy-terminal ubiquitin-associated (UBA) domain is required for them to function in cells [97-99]. This raises the intriguing question of whether dimerization of Cbl proteins allows them to detect and respond to the dimerization or clustering of receptor-tyrosine kinases upon activation.

## Catalytic efficiency and regulation through macromolecular juggling

In this review we have highlighted a few of the many impressive crystallographic studies delineating the large-scale conformational changes that underlie the catalytic action and regulation of ubiquitylation enzymes. E1 enzymes reorganize the three-dimensional arrangement of their domains to generate the active site environments for chemically distinct reactions and to progressively alter the affinities for their sequential macromolecular substrates. These features presumably allow E1 enzymes to achieve efficiency and directionality in the catalysis of multistep reactions. Similar mechanisms are likely to be used by enzymes in the HECT E3 family, which also rely on structural flexibility to catalyze multistep reactions. Moreover, conformational rearrangements are important in ubiquitylation enzymes that catalyze one-step reactions, as seen for members of the cullin-RING family. These multisubunit E3 enzymes re-orient individual subunits to allow the RING domain to approach target proteins of various sizes and to enable the formation of ubiquitin chains [100-102] (for reviews, see [27,28]). Structural studies on a particularly complex cullin-RING E3, the anaphase-promoting complex, are beginning to reveal how conformational changes in this giant, approximately 1.5 megadalton protein assembly affect function [103,104].

The need to efficiently process macromolecular substrates unites the various components of the ubiquitylation machinery, irrespective of their size and complexity. Unlike small metabolites that often interact with small surface crevices that can be opened or closed through relatively subtle structural fluctuations, protein substrates typically utilize large, flat surfaces to bind to enzymes. To modulate these surfaces and to actually juggle protein substrates without either holding on to them too long or dropping them prematurely presents a considerable challenge. Large-scale conformational rearrangements appear to have emerged as an evolutionary answer.

## Note

While this review was in press, Shaun Olsen and Christopher Lima published the crystal structure of a complex containing *Schizosaccharomyces pombe* E1 (Uba1), E2 (Ubc4), and ubiquitin that illuminates the structural basis of the final trans-thioesterification step in the catalytic cycle of canonical E1 enzymes [105].

## Abbreviations

PDB: Protein data bank.

## Supplementary Material

 Macromolecular juggling by ubiquitylation enzymes Click here for file
